# Comparative Transcriptome and Metabolic Profiling Analysis of Buckwheat (*Fagopyrum Tataricum* (L.) Gaertn.) under Salinity Stress

**DOI:** 10.3390/metabo9100225

**Published:** 2019-10-14

**Authors:** Weibo Ma, Jae Kwang Kim, Caihua Jia, Feifan Yin, Hyo Jin Kim, Waheed Akram, Xuebo Hu, Xiaohua Li

**Affiliations:** 1 Laboratory of Natural Medicine and Molecular Engineering, Department of Medicinal Plant, College of Plant Science and Technology, Huazhong Agriculture University, Wuhan 430070, China; weibo93_ma@163.com (W.M.); waheed.akram@mail.hzau.edu.cn (W.A.); xuebohu@mail.hzau.edu.cn (X.H.); 2 Division of Life Sciences and Bio-Resource and Environmental Center, Incheon National University, Yeonsu-gu, Incheon 406-772, Korea; kjkpj@inu.ac.kr (J.K.K.); kimhj6605@naver.com (H.J.K.); 3 Key Laboratory of Environment Correlative Dietology (Ministry of Education), College of Food Science and Technology, Huazhong Agricultural University, Wuhan 430070, China; chjia@mail.hzau.edu.cn; 4 Hubei Key Laboratory of Agricultural Bioinformatics, College of Informatics, Huazhong Agricultural University, Wuhan 430070, China; ffyin@webmail.hzau.edu.cn

**Keywords:** Tartary buckwheat, salinity stress, transcriptome, metabolic profiling, gene expression

## Abstract

Tartary buckwheat (*Fagopyrum tataricum* (L.) Gaertn.) is a nutritional crop, which has high flavonoid content. However, buckwheat is a salt sensitive glycophyte cereal crop and the growth and grain yield of buckwheat are significantly affected by soil salinity. In this study, we performed a comprehensive analysis of the transcriptome and metabolome of salt treated-buckwheat to understand the effects of salinity on buckwheat. A total of 50,681,938 clean reads were acquired from all samples. We acquired 94,950 unigenes with a mean length of 1133 bp and N50 length of 1900 bp assembly. Of these, 63,305 unigenes (66.7%) were matched in public databases. Comparison of the transcriptome expression patterns between control and salt treated groups showed that 4098 unigenes were up-regulated and 3292 unigenes were down-regulated significantly. Further, we found that genes involved with amino acid, lipid and nucleotide metabolism were most responsive to salt stress. Additionally, many genes involved in secondary metabolite biosynthesis changed significantly following treatment. Those affected included phenylpropanoid biosynthesis and flavonoid biosynthesis. Chromatographic analysis was used to examine the differences in concentration of flavonoids, carotenoids, amino acids and organic acids in the samples following treatment. There was a significant increase in rutin (12.115 mg/g dry weight), following salt stress; whereas, six carotenoids (lutein, zeaxanthin, 13Z-*β*-carotene, *α*-carotene, E-*β*-carotene and 9Z-*β*-carotene) did not significantly respond to salt stress. Ultimately, our data acts as a valuable resource for future research on buckwheat and can be used as the basis for future analysis focused on gene-to-metabolite networks in buckwheat.

## 1. Introduction

Salinity is one of the environmental factors that has the greatest effect on food production and quality. High salinity can limit the growth, development and yield of crops [1]. There are three salt-induced stress pathways—osmotic stress, ionic stress and secondary stress. High salinity can change salt and ion concentrations, with toxic effects and alterations to important metabolic pathways [2]. Most crops are salt sensitive, high Na+ concentration in the cytosol inhibits many metabolic and physiological processes [3]. High concentration of Na+ ions can also cause secondary stress in plant, including the accumulation of toxic compounds and disruption of nutrient balances [2]. Systematic research of salt-tolerance mechanisms has been reported in Arabidopsis, canola, rice and several others [4,5,6,7]. Previous studies have shown that canola responds to high salinity at the morphological, physiological and biochemical levels along with changes at the molecular level [4]. Secondary metabolites play an important role in the response to biotic and abiotic stresses in plants [8,9,10]. Many reports have shown that the synthesis of secondary metabolites, including carotenoids, anthocyanins and flavonoids, is heavily influenced by salt stress [11,12], these compounds could serve as the nonenzymatic scavengers to reduce reactive oxygen species (ROS) damage and protect cellular structures and macromolecules in plant [5,13,14].

Transcription factors play an important role in plant development and response to environment stress [15,16]. There are many transcription factors has been reported and involved in stress-responsive in plant. The transcription factor *TaMYB73* has been reported to as responding salinity stress improving ionic resistance partly via the regulation of stress-responsive genes in wheat [17]. *AtMYB20* was shown to negatively regulate type 2C serine/threonine protein phosphatases resulting in enhanced salt tolerance in *Arabidopsis* [18]. Zhong et al. reported two NAC transcription factors involved in response to high-salinity in *Brassica napus* [19]. Similar studies on the regulation of transcription factors in response to biotic or abiotic stress, including NAC, WRKY and bZIP amongst others, were also reported in rice, wheat, tomato and other plants [20,21,22,23,24].

Tartary buckwheat (*Fagopyrum tataricum* (L.) Gaertn., Polygonaceae family) is both a food and medicinal plant. It is predominantly grown in the semi-arid and arid regions of the mountainous areas of southwest China, India, Nepal, Japan and Korea [25,26]. The chemical composition of tartary buckwheat was comprehensively investigated, this included analysis of its flavonoid [27], phenylpropanoid glycosides [28], organic acids [29], production of various other important compounds [30,31]. In buckwheat, the major flavonoid is rutin, whose concentration can reach 81 mg/g in the groats of tartary buckwheat [32]. Rutin-rich buckwheat is known for reducing cholesterol, blood clots and high blood pressure [33,34,35,36], in addition to showing anti-oedema effects, antioxidant activity [37,38,39,40], anti-inflammatory activity [41] and antifatigue properties [42]. In addition, some proteins in buckwheat are believed to have anti-tumour properties [43,44,45].

The synthesis of secondary metabolites in buckwheat is strongly influenced by environmental factors. It has been reported that the content of flavonoids was mainly up-regulated and accumulated in buckwheat sprouts when treated with exogenous methyl jasmonate (MeJA) [46,47]. The accumulation of phenolic compounds and anthocyanins were obviously increased with the treatment of L-phenylalanine and light-emitting diodes (LEDs) lights in tartary buckwheat sprouts [48]. Buckwheat is a salt sensitive glycophyte cereal crop and the growth and grain yields are significantly affected by soil salinity [49,50]. In addition, the metabolic synthesis of buckwheat is also affected by salt stress. Gamma-aminobutyric acid (GABA) and flavonoid production are reported to alter when buckwheat sprouts are treated to high salinity conditions [51,52]. Two flavonol compounds, quercetin and kaempferol, were up-regulated in buckwheat cultivar “Hokkai T10” when treated with NaCl [53]. However, the comprehensive effect of salinity on transcriptional and metabolic activities in buckwheat has not been widely reported. [54].

In order to provide a global view of salinity-tolerant regulatory mechanisms in buckwheat, in this study, the influence of salt-induced salinity stress was investigated in buckwheat using transcriptome and metabolite profiling analysis. To do this at a molecular level, the Illumina HiSeq™ 2000 platform was employed to screen differential gene expression under salt stress. This data were then analyzed and the main metabolites, especially, flavonoids and carotenoids, were detected using chromatographic analysis. Our research provided foundational information which may lead to a better understanding of the molecular mechanisms of salt-resistance in buckwheat.

## 2. Results

### 2.1. De Novo Transcriptome Assembly of F. Tataricum and Sequence Analysis

We performed RNA-sequencing analysis using total RNA from plants exposed to normal and high salinity conditions. After removal of adaptor sequences and reads with low quality, a total of 50,681,938 clean reads were acquired from all samples. Subsequently, after assembling of these clean reads, 94,950 unigenes, with the average length of 1133 nucleotides (nt) and the N50 is 1900 nt, have been identified between control (C) and salt-treated (T) samples (Table 1). Among them, 42,127 unigenes have a length between 300 nt and 1000 nt and 15,263 unigenes are longer than 1000 nt (as shown in Supplementary Figure S1).

### 2.2. Functional Annotation and Classification of the Buckwheat Transcriptome

**Figure 1:**
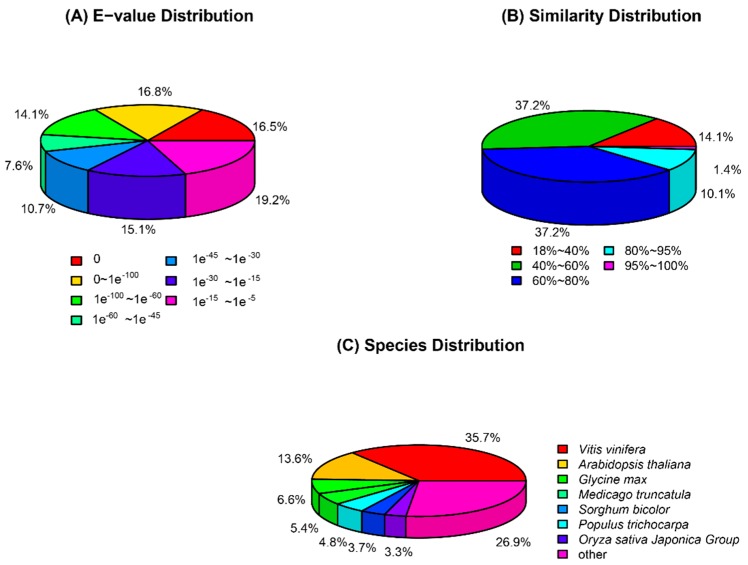
Species statistics and distribution of unigenes in *F. tataricum* transcriptome. (**A**) The E-value frequency distribution of Nr annotated unigenes using E-value frequency distribution analysis; (**B**) Sequence similarity distribution of the Blast hits for each unigenes; (**C**) The species homologous distribution analysis. It is shown as the percentage of the total homologous sequences in the Genebank non redundant (Nr) database.

To obtain gene function information, the assembled transcripts were subjected to a search through six public databases, including the NCBI non-redundant protein sequences database (Nr), the NCBI non-redundant nucleotide database (Nt), the Clusters of Orthologous Groups of protein databases (COG), the Swiss-Prot protein database (Swiss-Prot), the Kyoto Encyclopedia of Genes and Genomes (KEGG) database. Finally, 63,305 unigenes (66.67% of the assembled transcripts) were well annotated in at least one of the public databases (Supplementary Table S1, Figure S2). In detail, 60,148 unigenes were found in the Nr database, 52,067 in the Nt database, 43,129 in the Swiss-Prot database, 38,296 in the KEGG database and 28,107 in the COG database (Supplementary Table S1). Subsequently, using E-value frequency distribution analysis, 65.62% of the Nr annotated unigenes had an e-value < 1.0e^−30^ (Figure 1A). While sequence similarity distribution indicated that 48.50% of the unigenes showed more than 60% similarity with matched genes (Figure 1B). The species homologous distribution analysis of unigenes is shown in Figure 1C. These unigenes matched with the sequences from *Vitis vinifera* (21,470, 35.70%), *Arabidopsis thaliana* (8175, 13.59%), *Glycine max* (3.978, 6.61%), *Medicago truncatula* (3238, 5.38%) and *Sorghum bicolor* (2877, 4.78%) Figure 1C). The function of the unigenes from *F. tataricum* were divided into 3 major categories—‘biological process,’ ‘cellular component’ and ‘molecular function’ according to Gene Ontology (GO) analysis. Among the 57 sub-categories, the highest number of unigenes were putatively ascribed to involvement in the ‘cell’ (45,305), ‘cell part’ (45,017) and ‘organelle’ (39,256) under the category of cellular components. In the biological process category, the following were most strongly represented, ‘cellular process’ (40,743), ‘metabolic process’ (39,980) and ‘single-organism process’ (37,819) In the molecular function category enrichment for ‘binding’ (29,090) and ‘catalytic activity’ (28,351) was observed (Supplementary Figure S3). Classification of orthologous group (COG) analysis showed that 28,107 of the total transcripts could be placed in 25 categories. The top homologous categories were the cluster for ‘General function prediction only’ followed by ‘Transcription’ and ‘Replication, recombination and repair’ (Supplementary Figure S4).

### 2.3. Differentially Expressed Genes (DEGs) of Buckwheat in Response to Salt Stress

**Figure 2:**
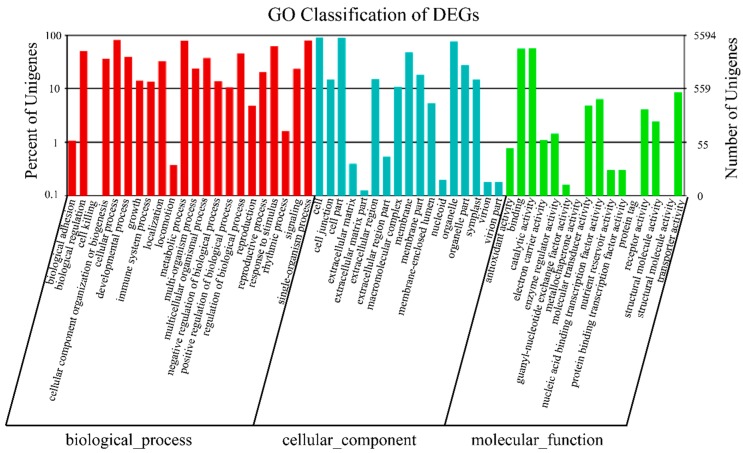
GO analysis of differentially expressed genes (DEGs) based on biological process, cellular component and molecular function categories.

**Figure 3:**
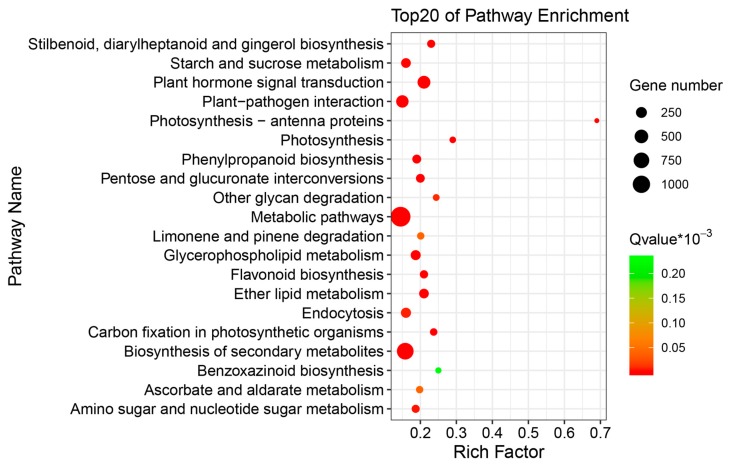
The differentially expressed genes (DEGs) with Kyoto Encyclopedia of Genes and Genomes (KEGG) pathway enrichment (top 20) in tartary buckwheat in response to salt stress.

The differentially expressed genes between salt treated and non-treated samples of buckwheat were analyzed. The results showed that significantly more transcripts (5350) were significantly up-regulated in salt treated samples than down-regulated (4207) when compared with control (*p*-value < 0.001 and |log2FoldChange| > 2). (Supplementary Figure S5). These differentially expressed genes were annotated using GO analysis and the results indicated that top clusters of differentially expressed genes (DEGs) belong to ‘cellular process,’ ‘single-organism process’ and ‘metabolic process’ in the biological process category. They fell into ‘cell,’ ‘cell part’ and ‘organelle’ in the cellular component category and ‘catalytic activity’ and ‘binding’ in the molecular function category (Figure 2). According to pathway annotation, the DEGs mainly fell into ‘Metabolic pathway’ (1414, 33.26%), ‘Biosynthesis of secondary metabolites’ (897, 21.1%) and ‘plant hormone signal transduction’ (419, 9.86%) ((Figure 3, Supplementary Table S2).

**Figure 4:**
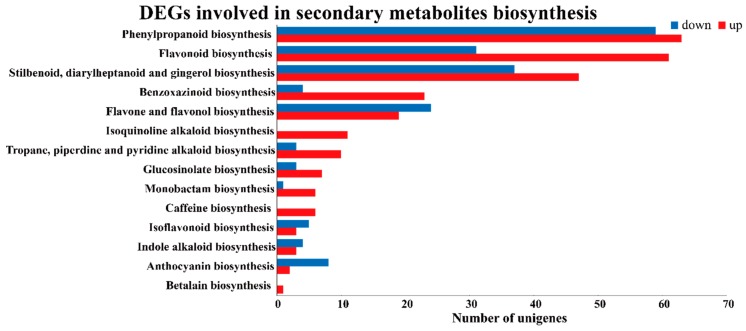
DEGs involved in secondary metabolites biosynthesis in buckwheat in response to salt stress.

According to GO analysis, 39,980 unigenes were linked to ‘metabolic processes,’ of which genes in ‘carbohydrates metabolism,’ ‘amino acid metabolism,’ ‘lipid and nucleotide metabolism’ were significantly differentially regulated under stress (Supplementary Figures S6 and S7, Table S2). In detail, 536 unigenes were up-regulated and 349 unigenes were down-regulated in carbohydrate metabolism under salt-stress (Supplementary Figure S6). Further, 167 unigenes from the starch and sucrose metabolism pathways were found to strongly respond to salt stress. The unigenes involved in glycolysis/gluconeogenesis, amino sugar and nucleotide metabolism, pyruvate metabolism, citrate cycle (TCA cycle), galactose metabolism, fructose and mannose metabolism were also predominantly up and not down regulated following salt stress (Supplementary Figure S6). While the unigenes involved in amino acid metabolism were largely up-regulated, especially in cysteine and methionine metabolism and the valine, leucine and isoleucine degradation process (Supplementary Figure S7). Secondary metabolites play an important role in stress resistance in plants. In our study, differentially expressed genes involved in 14 biosynthetic pathways were identified from the transcriptome data (Figure 4). The top salt-response genes belong to phenylpropanoid biosynthesis and flavonoid biosynthesis. In all, our results indicate that primary and secondary metabolic biosynthetic pathway were directly altered in response to salt-stress in buckwheat.

### 2.4. The Content of Metabolites in Tartary Buckwheat in Response to Salt Stress

**Figure 5:**
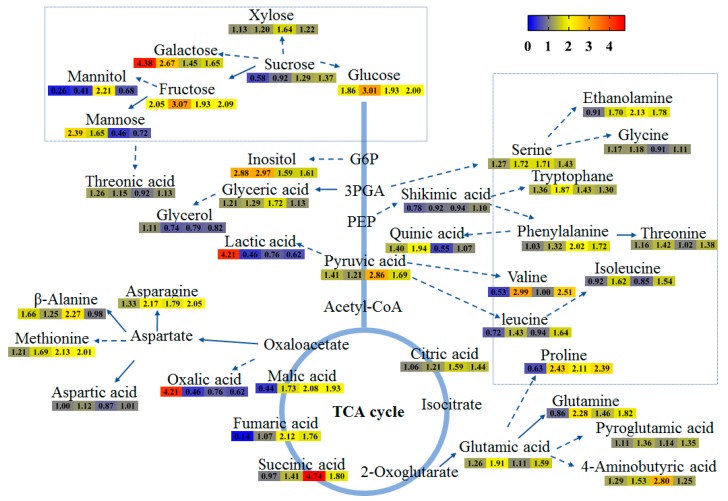
Overview of the primary metabolic changes of buckwheat in response to salt stress. The fold changes of each metabolite are given by salt-treated relative to control group in 1, 2, 4 and 5 day (cells from left to right, respectively). Different colors (from red, orange, yellow, grey yellow to blue) represent the fold change of the metabolites as indicated in the legend.

Primary metabolites, such as carbohydrates, amino acids, lipids and nucleotides, are all predominantly involved in physiological processes in living organisms. The comprehensive metabolic profiling of the primary metabolites was detected in tartary buckwheat in response to salt stress treatment. In our study, 39 hydrophilic metabolites and 14 liphophilic metabolites were identified as differentially regulated in buckwheat using GC-TOFMS, these included amino acids, organic acids and carbohydrates (Supplementary Tables S3 and S4). The sugars, including fructose, galactose, glucose, xylose and sucrose, content was increased in most of the salt-stress samples when compared with control. The level of galactose was 4.38- and 2.67-fold accumulated in the first two days treated samples, while, the content of glucose and fructose were 3.01-fold and 3.07-fold increased after the two days of treatment (Figure 5). Organic acids and amino acids including pyruvic acid, pyroglutamic acid, succinic acid and methionine, also showed a similar trend (Figure 5), indicating that they may contribute to the differential accumulation of secondary metabolites between salt-treated and control buckwheat samples. The concentration of lipophilic metabolites, including isoprenoid compounds α-tocopherol and *β*-sitosterol, had minor fluctuations, while no significant change was detected (Supplementary Tables S3 and S4).

**Figure 6:**
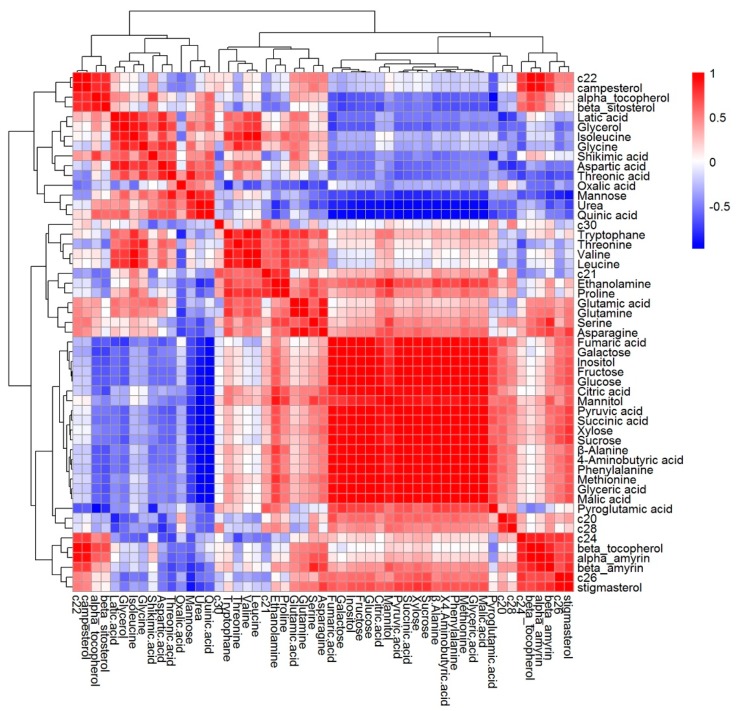
Correlation matrix and cluster analysis of results obtained from data on 53 primary metabolites of buckwheat. Each square indicates the Pearson’s correlation coefficient of a pair of compounds and the value for the correlation coefficient is represented by the intensity of the blue or red color, as indicated on the color scale.

Correlation analysis is a statistical method used to analyze the strength of the relationship between two quantitative samples; it can be applied to demonstrate the associations between metabolites belonging to specific biological systems [55]. To examine the relationship between lipophilic metabolites and hydrophilic metabolites in buckwheat, Pearson’s correlation analysis was performed (Figure 6). The most distinct relationships were observed between sugars and their derivatives (galactose, inositol, fructose, glucose, mannitol, xylose, sucrose), organic acids and the products of the tricarboxylic acid cycle (fumaric acid, malic acid, citric acid and succinic acid) and amino acids and their derivatives (*β*-alanine, phenylalanine, methionine, 4-aminobutyric acid).

### 2.5. The Transcriptional and Metabolic Regulation of Phenylpropanoid Biosynthetic Pathway of Buckwheat in Response to Salt Stress

**Figure 7:**
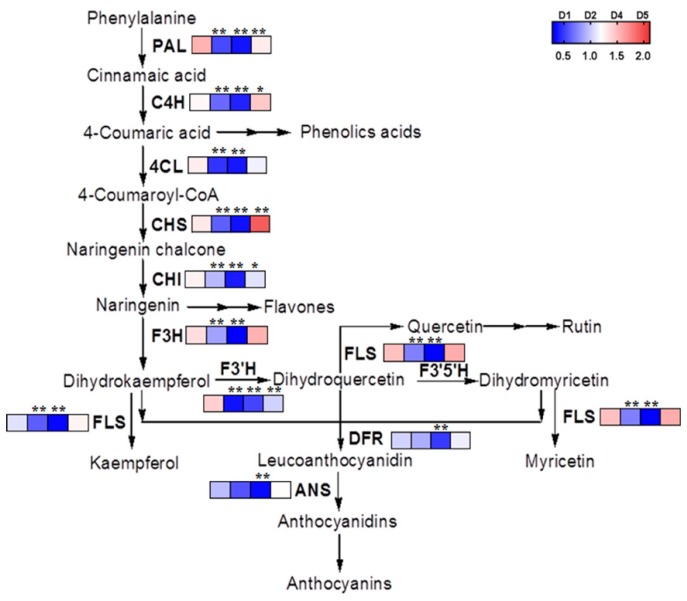
Expression of the key genes in phenylpropanoid biosynthetic pathway of tartary buckwheat in response to salt stress. PAL, phenylalanine ammonium lyase; CHI, chalcone isomerase; CHS, chalcone synthase; 4CL, 4-coumarate-CoA ligase; C4H, cinnamic acid 4-hydroxylase; F3H, flavanone-3-hydroxylase; F3′H, flavonoid-3′-hydroxylase; FLS, flavonol synthase; DFR, dihydroflavonol reductase; ANS, anthocyanin synthase. The asterisk indicates a significant difference between control (Con.) and salt-treated (Tr.) samples (*, *p* < 0.05; **, *p* < 0.01).

A total of 122 unigenes from phenylpropanoid biosynthesis were significantly differentially expressed between control and salt-treated (*p* < 0.01), of these, 63 unigenes were up-regulated and 59 were down-regulated (Supplementary Table S5). Interestingly, according to transcriptome data, a total of 92 unigenes from flavonoid biosynthesis were significantly differentially expressed under salt stress (*p* < 0.01), 61 of these unigenes were significantly up-regulated, while, only 31 were down-regulated (Supplementary Table S5). More unigenes in flavone and flavonol biosynthesis, isoflavonoid biosynthesis and anthocyanin biosynthesis pathway were down-regulated after salt stress when compared with the control (Figure 4, Supplementary Table S5). In this study, the gene expression profiles for the flavonoid biosynthesis pathway was established using quantification real-time PCR (qRT-PCR) (Figure 7). Our results showed that the main genes involved in flavonoid biosynthesis, including the genes from *FtPAL* to *FtRT*, were down-regulated after the first day of treatment and then significantly up-regulated in samples taken five days post treatment (Figure 7).

At the same time, we analyzed the main metabolites in buckwheat using high-performance liquid chromatography (HPLC). In total, eight main metabolites from flavonoid biosynthesis pathway, including catechin, chlorogenic acid, caffeic acid, *p*-coumaric acid, benzoic acid, rutin, quercetin, kaempferol, were detected and are shown in Table 2. The major metabolite in buckwheat, rutin, was significantly up-regulated in salt-treated samples, the majority of the rutin was accumulated during day one of treatment, where an increase of 12.24% was recorded when compare with the control. Rutin concentration initially decreased and then underwent significant upregulation in the following five-days post treatment, which was in agreement with the transcriptional data collected. Benzoic acid also showed a concentration spike at one day post treatment, while, catechin and quercetin were most enriched at four-days post treatment. Quercetin was 10-fold higher at four days post treatment when compared with the control. Other compounds did not show significant changes after salt treatment.

### 2.6. The Transcriptional and Metabolic Regulation of Carotenoid Biosynthesis of Buckwheat in Response to Salt Stress

Plant secondary metabolites, specifically carotenoids and phenolics, are stress inducible. These compounds play important roles in growth and contribute to the nutritional value of vegetables and crops [8]. Our transcriptome data shows that, 23 genes from the carotenoid biosynthesis pathway were up-regulated and 23 were down-regulated (Supplementary Table S6). As for the metabolites, 6 carotenoids including lutein, zeaxanthin, 13Z-*β*-carotene, α-carotene, E-*β*-carotene and 9Z-*β*-carotene were detected in this study, our data indicate that the predominant composition of carotenoids in tartary buckwheat seedlings were lutein and E-*β*-carotene, which was consistent with previous reports [56]. The accumulation of carotenoids changed with seedling age and the content of lutein, E-*β*-carotene and 13Z-*β*-carotene reached their highest level at four-days control group rather than salt-treated group. In detail, 299.29 μg/g and 249.16 μg/g of lutein, 235.54 μg/g and 164.47 μg/g of E-*β*- carotene, along with 47.27 μg/g and 37.24 μg/g of 13Z-β-carotene were detected at four days post treatment in the control and salt stress groups respectively. However, only zeaxanthin was found to increase significantly in four-days salt-treated buckwheat. Other carotenoids also did not show any significant accumulation following salt stress (Table 3).

## 3. Discussion

Plants have evolved sophisticated physiological adaptations to help them cope with various environmental stressors, including increased salinity [57]. This stress resistance is usually accomplished through various primary and secondary metabolites [8,10,12]. In previous reports, Jeon *et al*., reported that some organic acids, sugars and their derivatives, anthocyanins and proanthocyanidins were significantly increased in tartary buckwheat seedlings by cold treatment [58]. Anthocyanins accumulation was primarily detected in the epidermal and cortex cells of hypocotyls in tartary buckwheat sprout in response to cold stress [59]. According to metabolic analysis of wheat, five and eleven metabolites involved in glycolysis, TCA cycle and shikimate pathway, were significantly increased or decreased in the leaves of wheat seedlings during saline stress [60]. Various metabolites, including ascorbic acid, alkaloids, carotenoids, flavonoids, phenolics, tocopherol amongst others, have been reported as having expression profiles that are closely correlated with various stressors in plants, these metabolites mostly function as the nonenzymatic scavengers to protect plants from salt stress [9,12,61,62,63].

Transcriptomic analysis using next-generation sequencing (RNA-seq) has been successfully and widely applied to investigate the gene expression of plants and other organisms [64]. This technology has been used to elucidate the stress-response mechanisms in plants at the transcriptional level [2,4,65]. Lu *et al.* [54] reported that there was 385 DEGs with a fold change ≥ 2 and a false discovery rate (FDR) of <  0.05 in 15-days-old-seedlings in common buckwheat in response to salt stress (100 mM NaCl, 72 h). DEGs were highly enriched for ‘Protein processing in endoplasmic reticulum,’ ‘Plant hormone signal transduction,’ ‘Carbon metabolism,’ ‘Pentose phosphate pathway,’ ‘Biosynthesis of amino acids’ and ‘Citrate cycle (TCA cycle)’ [54]. Even the nutritution and biological characteristics are different from common buckwheat, the similar result was also detected in this tartary buckwheat research and the DEGs from control versus 100 mM NaCl treated groups mainly belong to ‘Metabolic pathway,’ ‘Biosynthesis of secondary metabolites,’ ‘Plant hormone signal transduction,’ ‘Starch and sucrose metabolism,’ ‘Amino sugar and nucleotide sugar metabolism’ and so on according to pathway annotation (Figure 3, Table S2); this result indicated that the primary and secondary metabolites biosynthetic pathway were obviously activated at the transcriptional level in buckwheat in response to salt stress.

According to the transcriptome data, there were a number of transcripts (5350 up- and 4207 down-regulated) differentially regulated following salt treatment (*p*-value < 0.001 and |log2FoldChange| > 2) (Figure S5). A total of 683 unigenes were annotated, 432 were up-regulated and 251 down-regulated in buckwheat during salt stress. These results reveal that more genes are involved in buckwheat in response to salt stress than previous studies had identified [66]. In the previous study 14-day-old Tartary buckwheat seedlings (Chuanqiao No.1) were exposed to 200 mM NaCl for 24 hours. Here they identified 455 DEGs, these DEGs were mainly involved the biochemical pathways including ‘Antigen processing and presentation,’ ‘Protein processing in endoplasmic reticulum,’ ‘Estrogen signaling pathway’ and ‘Plant-pathogen interaction’ when analyzed using KEGG enrichment analysis [66]. In contrast, the DEGs identified in this study predominantly featured in the following pathways ‘Metabolic pathways’ (33.26%), ‘Biosynthesis of secondary metabolites’ (21.1%) and ‘plant hormone signal transduction’ (9.86%) (Figure 3, Table S2). These differences may be the result of differences in the cultivars, seedling age of buckwheat and the severity and timing of the salt stress [66]. According to previous reports, the accumulation of primary or secondary metabolites induced by salinity stress may vary and base on different species, cultivars or treatment way [10,11,53]. It has been reported that the phenolic acids are species-specific among Brassicaceae and some phenolic acids may especially participate in stress tolerance in more salt-tolerant varieties or species, such as white cabbage and kale [67]. Interestingly, the NaCl treatment lead to different increased degree of carotenoids in one nonanthocyanin-accumulating and three anthocyanin-accumulating genotypes tomato, however, the accumulation of total anthocyanins was opposite in different tomato cultivars [11]. The result also suggested that a considerable number of genes and metabolites were differentially regulated in salt-tolerant or not tolerant varieties or species. This further indicated that it is important to study and exploit the viability of saline soil suitable buckwheat species or cultivars with increased levels of functional compounds.

Combining transcriptomic and metabolic data is one approach to understanding the complex biological processes involved in plants [68,69,70,71]. Using transcriptomic and metabolic analysis, Jeon *et al*., reported that the metabolites including some organic acids, sugars and their derivatives, anthocyanins and proanthocyanidins were significantly increased and some amino acids and their derivatives were decreased in tartary buckwheat seedlings by cold treatment and the transcription levels of genes in phenylpropanoid biosynthetic pathway were correspondingly accumulated in response to cold stress [58]. Even some researchers reported that salinity effected the accumulation of phenolic compounds, carotenoids and flavonoids in buckwheat and the salt-related genes were analyzed by de novo assembly method [54,66], however, the interactive effects of salinity on both transcriptional and metabolic regulation of buckwheat was unclear [53,54,66,72]. In this study, we investigated the impact of salt stress on the transcriptional and metabolism response of buckwheat through combining transcriptomic and metabolic profiling approach. The content of primary and secondary metabolites, including carbohydrates, organic acids or amino acids, flavonoids, carotenoids and tocopherols were detected, while the accumulations of above metabolites were differentially modulated in buckwheat in response to salt stress treatment.

According to our results, most of the sugars, organic acids and amino acids increased during salt-stress, which was a little different from the accumulation pattern of metabolites in response to cold stress [58]. This result further indicated that environment stress could affect the nutritional constituents of buckwheat, on the other side, buckwheat may modulate the metabolites accumulation to increase tolerance to environmental stress [46,58,72]. In addition, this metabolites result was consistent with the transcriptomic data, in which DEGs in ‘metabolic pathways’ (33.26%), ‘carbohydrate metabolism,’ ‘amino acid metabolism,’ ‘lipid metabolism’ and ‘nucleotide metabolism’ underwent significant expressional changes during salt stress (Figures S6 and S7). Flavonoids are the major secondary metabolites with diverse biological activities in *F. tataricum*. According to the transcriptomic data, 61 unigenes in flavonoid biosynthesis were up-regulated and 31 unigenes were down-regulated following salt treatment (Table S5). Correspondingly, metabolic analysis showed massive accumulation of rutin following salt stress, the result was corresponded with the most of genes expression level of rutin biosynthesis pathway in buckwheat. Carotenoids biosynthetic metabolites plays a major role in photosynthesis, which is one of the most severely affected pathways in plants response to salt stress [6,73]. Here, 23 genes from the carotenoid biosynthesis pathway were up-regulated and 23 down-regulated, however, the content of the six main carotenoids were not strongly affected by salt stress, which may be the result of the limited treatment course as discussed above. Other factors also could affect the carotenoids synthesis in buckwheat, it has been reported that the light-grown buckwheat sprouts exhibited higher and increased content of carotenoids during the mid-cultivation when compared with dark condition, while, the decreased trend was monitored under the longer culture time, the accumulation pattern of carotenoid was also fluctuated with the seed development stages, different organs and cultivars of tartary buckwheat [56].

**Figure 8:**
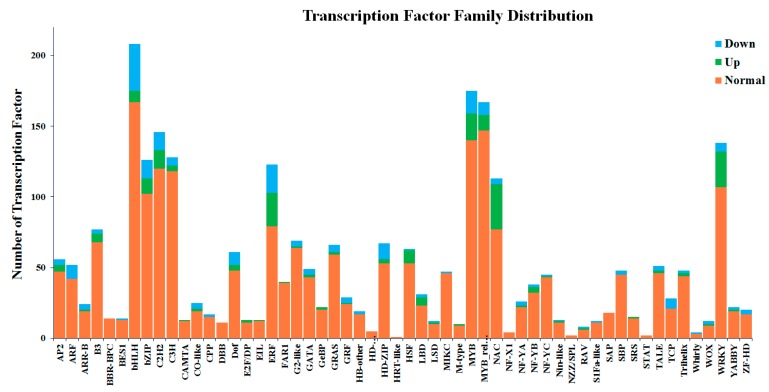
The differentially expressed transcription factors (TFs) of buckwheat in response to salt stress. The up and down regulated TFs were represented by the green or blue color, respectively.

In addition, it has been shown that many transcription factors (TFs), including NACs, MYBs and WRKYs, are involved in the regulation of the salt stress response in plants [74,75]. *TaMYB56-B* in wheat and *MdSIMYB1* in apple were reported to be involved in the salt stress response [76] and the over-expression of *TaMYB73, AtMYB20* and *OsMYB3R-2* in *Arabidopsis* can enhance salt tolerance [17,18,77]. It has been reported that eight R2R3-MYB genes from buckwheat serve a functional role in abiotic stress tolerance [78]. *FtMYB10* from buckwheat has been reported to act as a negative regulator of salt and drought stress, this mechanism has been linked to ABA signaling feedback regulation [79]. Our study has identified a number of transcription factors, including NAC, bHLH and MYB genes, which respond to salt stress in buckwheat seedlings. Of the 2664 identified transcription factors, the bHLHs, ERFs, MYBs, NACs and WRKYs, were significantly differentially regulated during salt stress. Totally, 30 and 25 MYBs and MYBs-related genes, 32 and 4 NACs, 8 and 33 bHLHs, 25 and 6 WRKYs were up or down-regulated to response to salt stress, respectively (Figure 8, Table S7), which may play an important role in regulating salt stress in buckwheat. It has been demonstrated that transcription factors are involved in modulating metabolic pathways in response to abiotic or biotic stressors [20,21,77]. Understanding these processes at the molecular level will play an important role in stabilizing crop performance under high salt conditions [65].

## 4. Materials and Methods 

### 4.1. Plant Materials, Culture Conditions and Salinity Treatment

*F. tataricum* seeds (Yu Qiao 2) were germinated and cultured in soil under light/dark (16/8 h,13000 flx) conditions at 25 °C with approximately 50% humidity. 10-days-old buckwheat seedlings were treated with exogenously NaCl solution (100 mM). The control and salt-treated samples were harvested after 1, 2, 4 and 5 days without roots. Samples were rapidly frozen in liquid nitrogen after collection and stored at −80 °C until analysis.

### 4.2. RNA Isolation, Library Preparation and Transcriptome Sequencing

For the transcriptome sequencing analysis, the 4 days samples of three repeats were precooled and ground into powder in a mortar using liquid nitrogen. Total RNA was extracted from samples using a Trizol reagent (Invitrogen). Finally, the purified double-stranded cDNA samples were further enriched by polymerase chain reaction (PCR) to construct the final cDNA libraries that were sequenced using Hiseq 2000 (Illumina Technologies) to generate 150 bp paired ends.

### 4.3. Illumina Sequencing and De Novo ASSEMBLY

The raw reads were filtered low quality and removal of adapter sequences, then get clean reads do de novo assembly by Trinity software package [80]. The Trinity program combines overlapping reads into longer contig, through the relationship between reads. Then confirm different contig from same sample and each distance, connect those contig to get unigenes. In order to annotate the assembled transcripts, blast searches were performed against databases including the Nr, Nt, Swiss-Prot, KEGG and COG with the threshold of e-value < 0.00001), along with function annotation of each sequence. The GO annotations were using Blast2GO program and using WEGO software for gene function distributions. The genome information of buckwheat was used as reference information to annotate the transcripts data [81].

### 4.4. Availability of Supporting Data

The sequencing data in this study were stored in the NCBI Sequence Read Archive (SRA, http://www.ncbi.nlm.nih.gov/Traces/sra/) with the accession number of PRJNA528524.

### 4.5. Total RNA Extraction and cDNA Synthesis

Total RNA was isolated from *F. tataricum* frozen samples using the RNeasy Plant Mini Kit (Qiagen, Valencia, CA, USA). First-strand cDNA was synthesized from 1 μg of high-quality total RNA with a ReverTra Ace-R reverse transcription (RT) kit using oligo(dT)18 primer (Toyobo Co., Ltd., Osaka, Japan). A 20-fold dilution of 20 μL of the resulting cDNA was used as a template for quantitative real-time PCR.

### 4.6. Quantitative Real-Time PCR (qRT-PCR) Analysis

The quantitative real-time PCR (qRT-PCR) was performed and the primers were designed and used according to previous reference [82]. The templates were amplified under following conditions—95 °C for 5 min, followed by 35 cycles at 95 °C for 15 s, annealing for 15 s at 56 °C and elongation for 20 s at 72 °C. Real-time PCR reactions were run on a MiniOpticon system (Bio-Rad Laboratories, Hercules, CA, USA) using the SYBR Green Real-Time PCR Master Mix (Toyobo, Osaka, Japan). The housekeeping gene *FtActin* (KC571237) was used as the internal control to normalize gene expression, as described previously [56]. All reactions were performed in triplicate, addition each run contained a series of standards and a negative control (using water instead of cDNA). The relative expression levels were calculated as differences in cycle threshold (Ct) between genes and actin and analyzed using the 2^−△△Ct^ method [83].

### 4.7. Extraction and Quantitative HPLC Analysis for Flavonoids

The materials were freeze dried and then ground to a powder before extraction using a method described previously with some modifications [53]. 0.10 g of each powdered sample was extracted with 5 mL of 80% methanol (MeOH), after extraction with sonicator at 25 °C for 1 h, the centrifugation was performed at 12,000 × *g* for 10 min, the supernatant was filtered and then used for analysis. The HPLC analysis of flavonoids was performed on a Shimadzu class-VP HPLC system (Kyoto, Japan) equipped with a Capcell PAK ODS column (250 mm × 4.6 mm, 5 μm; Shiseido, Tokyo, Japan). The mobile phase consisted of (A) MeOH and (B) water—0.1% formic acid (*v/v*). The gradient condition was as follows—0 min, 95% A; 4 min, 95–85% A; 9 min, 85% A; 14 min, 85–80% A; 24 min, 80% A; 54 min, 80–70% A; 55 min, 70–55% A; 65 min, 55% A; 75 min, 55–44% A; 77.0 min, 44–40% A; 79 min, 40% A; 80 min, 40–20% A; 90 min, 20% A; 91.0 min, 20–95% A; and 98.0 min, 95% A. The column was maintained at 30 °C, the flow rate was 1.0 mL/min, the injection volume was 20 μL and the detected wavelength was 280 nm. Each compound was quantified according to the peak areas. Results were expressed as milligrams per gram dry weight.

### 4.8. Liphophilic and Hydrophilic Metabolites Analysis Using GC-TOF-MS

The extraction and analysis of hydrophilic and liphophilic metabolites were performed as described previously [55,70,84,85]. For extraction of hydrophilic compounds, a total of 20 mg of ground sample was extracted with 1 mL of a mixed solvent of methanol/water/chloroform (2.5:1:1, *v/v/v*). Ribitol (120 μL, 0.2 mg/mL) was added as an internal standard (IS). Extraction was performed at 37 °C using a thermomixer compact (Eppendorf AG, Germany). For methoxime derivatization, 80 μL of methoxyamine hydrochloride (20,000 ppm) in pyridine was added and shaken for 90 min at 30 °C. After the addition of 80 μL of N-methyl-N-trimethylsilyltrifluoroacetamide (MSTFA), the mixtures were incubated for 30 min at 37 °C. The metabolites were analyzed by GC-TOF-MS using an Agilent 7890A gas chromatograph (Agilent, Atlanta, GA, USA) coupled to a Pegasus HT TOF mass spectrometer (LECO, St. Joseph, MI). Derivatized sample (1 μL) was separated on a fused-silica capillary column (30 m × 0.25 mm id) coated with 0.25 mm of CPSIL 8 CB low bleed (Varian Inc., Palo Alto, CA, USA). The parameters of instrument and temperature program was set as mentioned in reference [70]. The quantification of each compounds was based on the peak area ratio relative to that of the IS [55,70]. To extract the lipophilic metabolites, fine-ground samples (0.05 g) were added to 3 mL of ethanol containing 0.1% ascorbic acid (*w/v*) and 5 µL of 5 α-cholestane (100 µg/mL), which acted as IS and placed in a water bath at 85 °C for 5 min [84]. The mixture was added to 120 µL potassium hydroxide (80%, *w/v*) for saponification. The samples were then directly treated in ice for 5 min and 1.5 mL of deionized water and hexane were added to each sample and this was mixed for 20 s by vortex and centrifugation (1200× g, 5 min, 4 °C). The supernatant was separated in fresh tubes. The pellet was subjected to re-extraction using 1.5 mL hexane. The hexane fraction was evaporated under a stream of N_2_ to a volume of 200 µL and dried in a centrifugal concentrator (CC-105). For derivatization, 30 µL of MSTFA and 30 µL of pyridine were added to each sample and the mixture was incubated (60 °C, 30 min, 1200 rpm) using a Thermomixer Comfort. The samples (1.0 µL each) were injected in split mode (10:1 ratio) and gas chromatography was performed using an Rtx-5MS column (30 m length, 0.25 mm diameter and 0.25 µm thickness). The GCMS-QP2010 Ultra system, with autosampler AOC-20i (Shimadzu), was used for the separation of the lipophilic compounds. The parameters of the instrument and temperature program were set as mentioned in References [84,85]. Calibration samples were prepared by mixing appropriate aliquots of each stock solution. Quantification was performed by means of three-point calibration curves, for which the concentrations of a mixture of authentic standards ranged from 0.25 to 5.0 µg, whereas the amount of the IS was constant at 0.5 µg [84,85].

### 4.9. Carotenoid Extraction and Analysis

Carotenoids were extracted and measured using HPLC as described previously [56]. Briefly, the carotenoids were extracted from cauliflower samples (0.1 g) by adding 3 mL of ethanol containing 0.1% ascorbic acid (*w/v*), vortex mixing for 20 s and placing in a water bath at 85 °C for 5 min. The potassium hydroxide (120 μL, 80% *w/v*) was used for carotenoid saponification (85 °C for 10 min). The cold deionized water (1.5 mL) was added after saponification. *β*-Apo-8′-carotenal (0.2 mL, 25 μg/mL) was added as an internal standard (IS). The carotenoids were extracted twice with 1.5 mL hexane and followed by centrifugation at 1200g. Aliquots of the extracts were dried under a stream of nitrogen and redissolved in 50:50 (*v/v*) dichloromethane/methanol before HPLC analysis. The samples were analyzed by an Agilent 1100 HPLC instrument (Massy, France) equipped with a photodiode array detector. The C30 YMC column (250 × 4.6 mm, 3 μm; YMC Co., Kyoto, Japan) was used. The wavelength of monitor was 450 nm. Solvent A consisted of methanol/water (92:8 *v/v*) with 10mM ammonium acetate; solvent B consisted of 100% methyl tert-butyl ether. The elution rate was 1 mL/min with a gradient program to analyze samples. Gradient elution was performed as follows—0 min, 90% A–10% B; 20 min, 83% A–17% B; 29 min, 75% A–25% B; 35 min, 30% A–0% B; 40 min, 30% A–70% B; 42 min, 25% A–75% B; 45 min, 90% A–10% B; and 55 min, 90% A–10% B. The quantification of individual carotenoid was calculated with carotenoid standards (Carote Nature, Lupsingen, Switzerland) according to the peak area ratios with internal standard (IS).

### 4.10. Data Processing and Statistical Analysis

All analyses were performed at least three times. Experimental data were processed by analysis of variance (ANOVA) and significant differences among the means were determined by Duncan’s multiple-range test (SPSS 22.0, Chicago: SPSS Inc. IL, USA). Pearson correlation analysis was calculated by SPSS 22.0, then cluster analysis and heatmap were drawn by R (http://www.r-project.org/).

## 5. Conclusions

To our knowledge, no previous studies have provided a comprehensive description of salt-resistance in buckwheat using transcriptome and metabolite profiling analysis and the interactive effects of salinity on both transcriptional and metabolic regulation of buckwheat was unclear. In this study, we performed a comprehensive analysis of the transcriptome and metabolome of salt treated-buckwheat to understand the effects of salinity on buckwheat. Many genes and metabolites involved in primary and secondary metabolic biosynthesis were changed significantly following treatment. Those effects especially included amino acids and organic acids biosynthesis, phenylpropanoid biosynthesis and flavonoid biosynthesis in buckwheat. In all, this research illuminated more information to uncover the regulation mechanism of salt tolerance in buckwheat at the transcriptional and metabolic level. Our study could be helpful for the better understanding of the salt tolerance mechanisms in this important crop.

## Figures and Tables

**Table 1:** Statistic of sequencing and de novo assembling of transcriptome in *F. tataricum*.

	Sample	Total Number	Total Length (nt)	Mean Length (nt)	N50	Total Consensus Sequences	Distinct Clusters	Distinct Singletons
**Contigs**	C1	97,017	48,783,474	503	1133	-	-	-
	C2	103,961	51,398,910	494	1104	-	-	-
	C3	98,588	49,113,021	498	1130	-	-	-
	T1	117,182	52,926,262	452	1007	-	-	-
	T2	104,429	49,990,958	479	1081	-	-	-
	T3	118,868	53,985,959	454	1001	-	-	-
**Unigenes**	C1	58,789	60,570,998	1030	1680	58,789	21,105	37,684
	C2	62,559	64,289,688	1028	1686	62,559	22,445	40,114
	C3	58,644	60,081,202	1025	1668	58,644	20,637	38,007
	T1	64,470	62,513,110	970	1639	64,470	21,291	43,179
	T2	60,146	60,524,742	1006	1675	60,146	20,498	39,648
	T3	65,749	63,160,040	961	1631	65,749	20,948	44,801
	All	94,950	107,581,109	1133	1900	94,950	38,242	56,708

C1-3 and T1-3 mean control (C) and salt-treated (T) samples with three repeats, respectively.

**Table 2:** The contents of flavonoids in tartary buckwheat in response to salt stress (μg/g dry weight).

Samples		Catechin	Chlorogenic Acid	Caffeic Acid	*p*-Coumaric Acid	Benzoic Acid	Rutin	Quercetin	Kaempferol
**Con.**	1D	12.04 ± 2.36a	625.66 ± 28.28cd	46.32 ± 0.03ab	12.73 ± 2.72abc	176.59 ± 8.75c	12115.99 ± 132.05f	54.84 ± 10.24c	34.28 ± 4.42bc
	2D	11.56 ± 0.72a	596.23 ± 3.06c	37.29 ± 6.07ab	14.2 ± 2.06bc	162.52 ± 8.19b	10406.33 ± 41.03c	36.55 ± 14.71bc	32.74 ± 6.92ab
	4D	19.21 ± 1.99bc	776.19 ± 26.62d	49.46 ± 0.02b	10.32 ± 0.72a	144.29 ± 3.23a	9506.46 ± 212.82a	10.52 ± 4.88a	39.45 ± 1.21bc
	5D	14.61 ± 0.07ab	535.18 ± 18.28ab	38.83 ± 1.13ab	10.24 ± 0.27a	148.43 ± 4.77a	9906.37 ± 78.64b	52.76 ± 6.32c	26.81 ± 1.09a
**Tr.**	1D	13.62 ± 5.58ab	543 ± 31.06b	48.32 ± 5.86b	15.85 ± 2.44c	200.01 ± 3.07d	13599.51 ± 112.49g	22.76 ± 4.03ab	38.02 ± 4.34bc
	2D	21.66 ± 2.00c	516.64 ± 10.10ab	44.26 ± 0.43ab	10.61 ± 0.21ab	184.06 ± 2.38c	11743.61 ± 110.46e	46.73 ± 19.37c	32.67 ± 1.83ab
	4D	23.04 ± 3.32c	639.35 ± 6.33c	39.99±14.99ab	11.6 ± 0.48ab	146.85 ± 1.43a	9975.95 ± 93.02b	111.04 ± 11.74d	41.14 ± 5.04c
	5D	13.47 ± 5.87ab	503.38 ± 1.84a	33.46 ± 9.32a	13.07 ± 3.55abc	167.23 ± 5.67b	11370.11 ± 0.01d	47.5 ± 15.36c	37.12 ± 1.94bc

Values with different letters indicate a significant difference between control (Con.) and salt-treated (Tr.) samples at *p* < 0.05, applying a Duncan test (SPSS 22.0).

**Table 3:** The contents of carotenoid in tartary buckwheat in response to salt stress (μg/g dry weight).

Sample		Lutein	Zeaxanthin	13Z-*β*-Carotene	*α*-Carotene	E-*β*-Carotene	9Z-*β*-Carotene
**Con.**	1D	244.38 ± 7.96a	1.01 ± 0.25a	38.58 ± 4.24a	5.46 ± 0.46a	193.78 ± 9.61b	26.63 ± 1.88ab
	2D	236.83 ± 7.12a	1.16 ± 0.19a	36.34 ± 1.91a	4.32 ± 0.59a	172.21 ± 12.27ab	22.57 ± 0.73a
	4D	299.29 ± 20.97b	2.68 ± 0.41d	47.27 ± 2.02b	4.88 ± 0.09a	235.54 ± 25.51c	29.11 ± 3.31b
	5D	225.37 ± 17.38a	1.80 ± 0.15bc	33.19 ± 1.53a	4.61 ± 0.72a	156.30 ± 14.33a	21.86 ± 2.42a
**Tr.**	1D	242.01 ± 14.83a	1.42 ± 0.25abc	38.46 ± 0.67a	4.86 ± 0.29a	179.58 ± 19.13ab	25.89 ± 2.49ab
	2D	253.28 ± 36.49a	1.27 ± 0.13ab	37.51 ± 5.69a	5.11 ± 1.57a	177.76 ± 25.53ab	26.02 ± 6.26ab
	4D	249.16 ± 31.94a	3.24 ± 0.55e	37.24 ± 1.20a	5.54 ± 0.19a	164.47 ± 23.28ab	29.88 ± 3.85b
	5D	241.95 ± 9.48a	1.89 ± 0.22c	34.61 ± 0.52a	5.02 ± 0.06a	157.60 ± 11.81a	26.12 ± 2.43ab

Values with different letters indicate a significant difference between control (Con.) and salt-treated (Tr.) samples at *p* < 0.05, applying a Duncan test (SPSS 22.0).

## Supplementary Materials

The following are available online at https://www.mdpi.com/2218-1989/9/10/225/s1, Figure S1: Length distribution of all unigenes from F. tataricum transcriptome. Figure S2: Annotation of all unigenes from F. tataricum transcriptome. Number of genes were annotated by five representatively databases (Nr, KEGG, COG, Swissprot and Nt). Figure S3: GO analysis of all unigenes based on biological process, cellular component and molecular function categories. Figure S4: COG function classification of all unigenes sequences from F. tataricum transcriptome. Figure S5: Distribution of differentially expressed genes (DEGs) between control and salt treated buckwheat. DEGs was selected by p-value < 0.001 and |log2FoldChange| > 2. Figure S6: DEGs involved in carbohydrate metabolism between control and salt treated buckwheat. Figure S7: DEGs involved in amino acid metabolism between control and salt treated buckwheat. Table S1: Statistics of annotations for assembled unigenes of F. tataricum in different public databases. Table S2: DEGs genes with KEGG pathway annotation. Table S3: The relative comparation of hydrophilic metabolites in control (Con.) and salt-treated (Tr) buckwheat samples. Table S4: The content of liphophilic metabolites in control (Con) and salt-treated (Tr) buckwheat samples (μg/g dry weight). Table S5: Different expression of phenylpropanoid biosynthetic genes under salt stress. Table S6: The differentially expressed genes of carotenoids biosynthetic pathway in buckwheat under salt stress. Table S7: Identification of differentially expressed transcription factors (TFs) in buckwheat to response salt stress.

## Abbreviations

GO	Gene ontology
KEGG	Kyoto Encyclopedia of Genes and Genomes
KOG	Eukaryotic Ortholog Groups
Nr	NCBI non-redundant protein sequences
Nt	NCBI non-redundant nucleotide sequences
DEGs	Differential expressed genes
HPLC	High-performance liquid chromatography
GC-TOF-MS	Gas Chromatography Time-Of-Flight Mass Spectrometry
