# Supplementary material for: Comparative Transcriptome and Metabolic Profiling Analysis of Buckwheat (Fagopyrum Tataricum (L.) Gaertn.) under Salinity Stress
Source: Metabolites. 2019 Oct 14;9(10):225. doi: 10.3390/metabo9100225 (PMC6835380; doi:10.3390/metabo9100225)
Supplement: Supplementary file 1 [file metabolites-09-00225-s001.zip › Supplemental materials10-13/bw-nacl-supplementary figures.docx]

**Supplementary files**

**Supplementary Figure S1.**

**
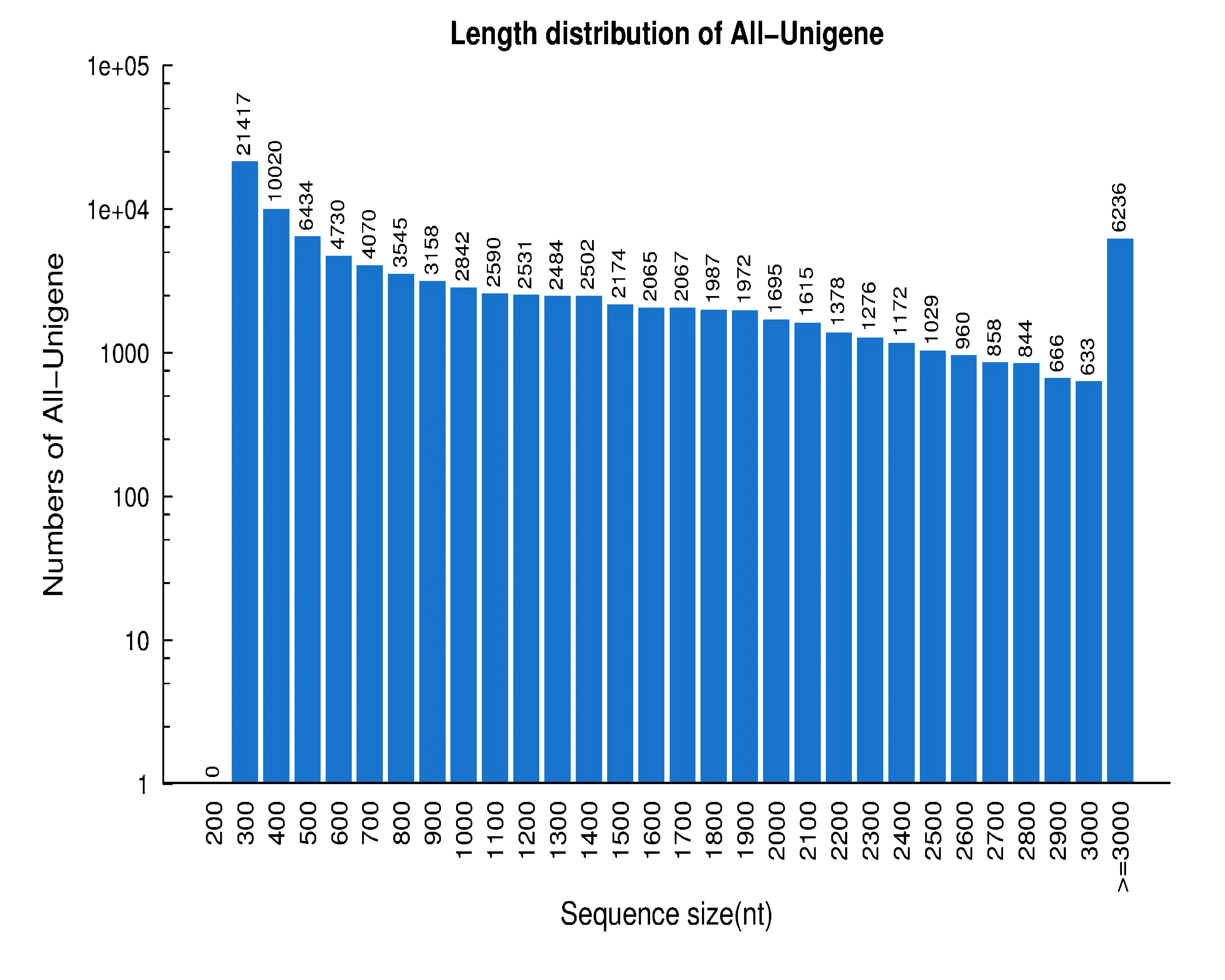
**

**Supplementary Figure S2.**

**
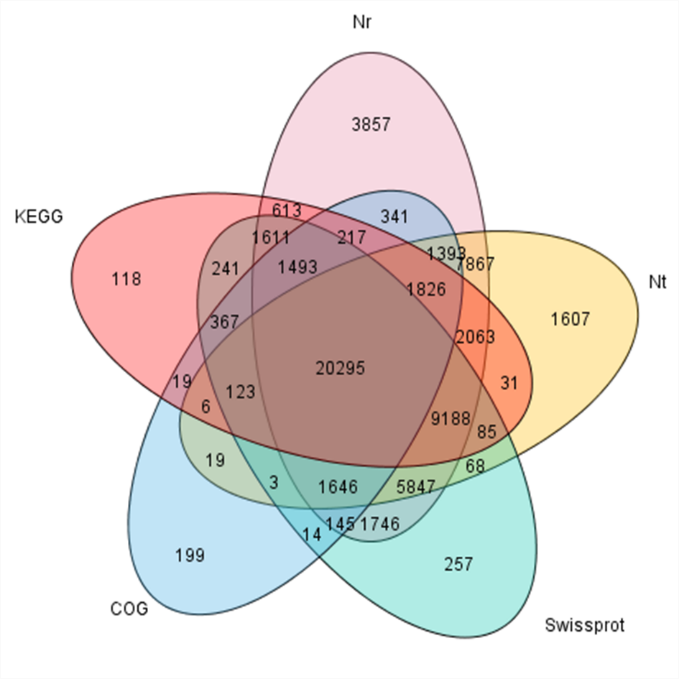
**

**Supplementary Figure S3.**

**
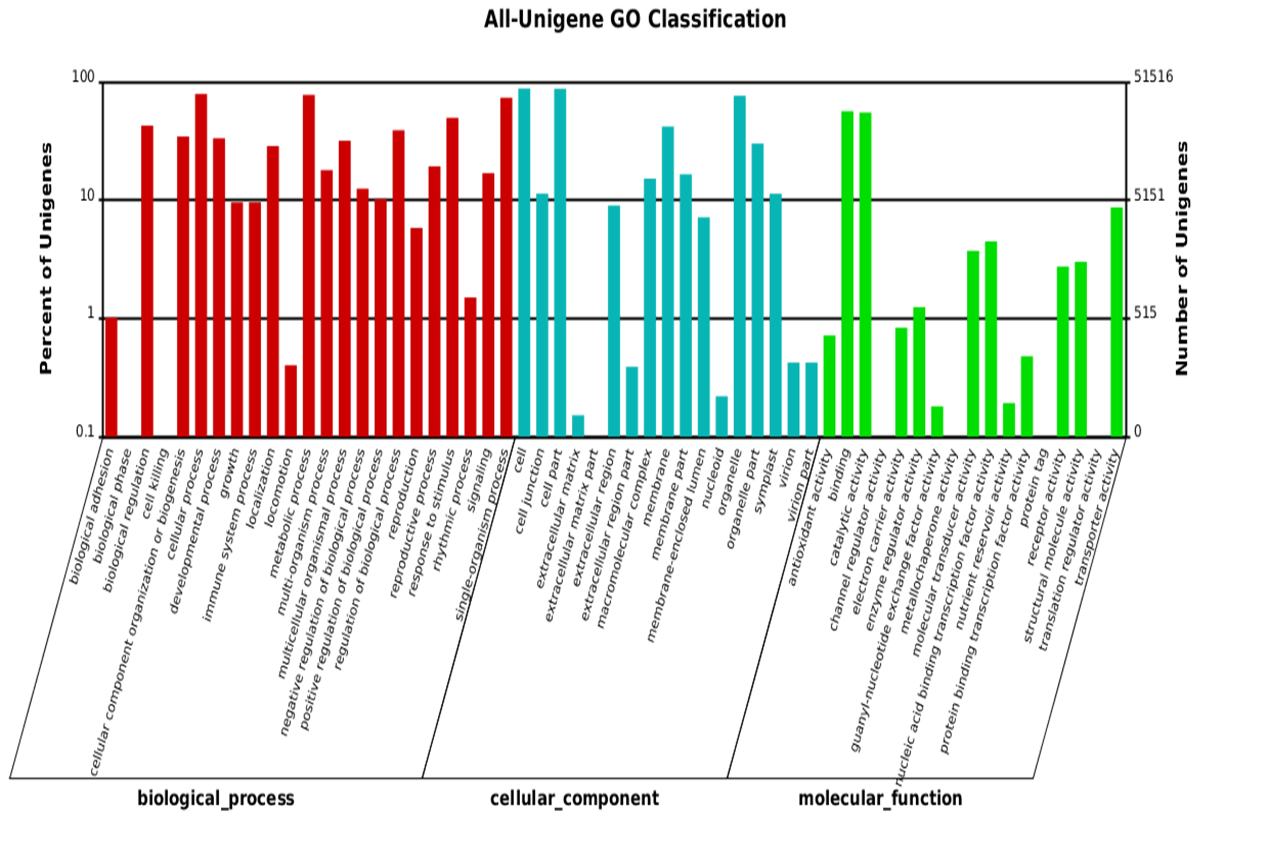
**

**Supplementary Figure S4.**

**
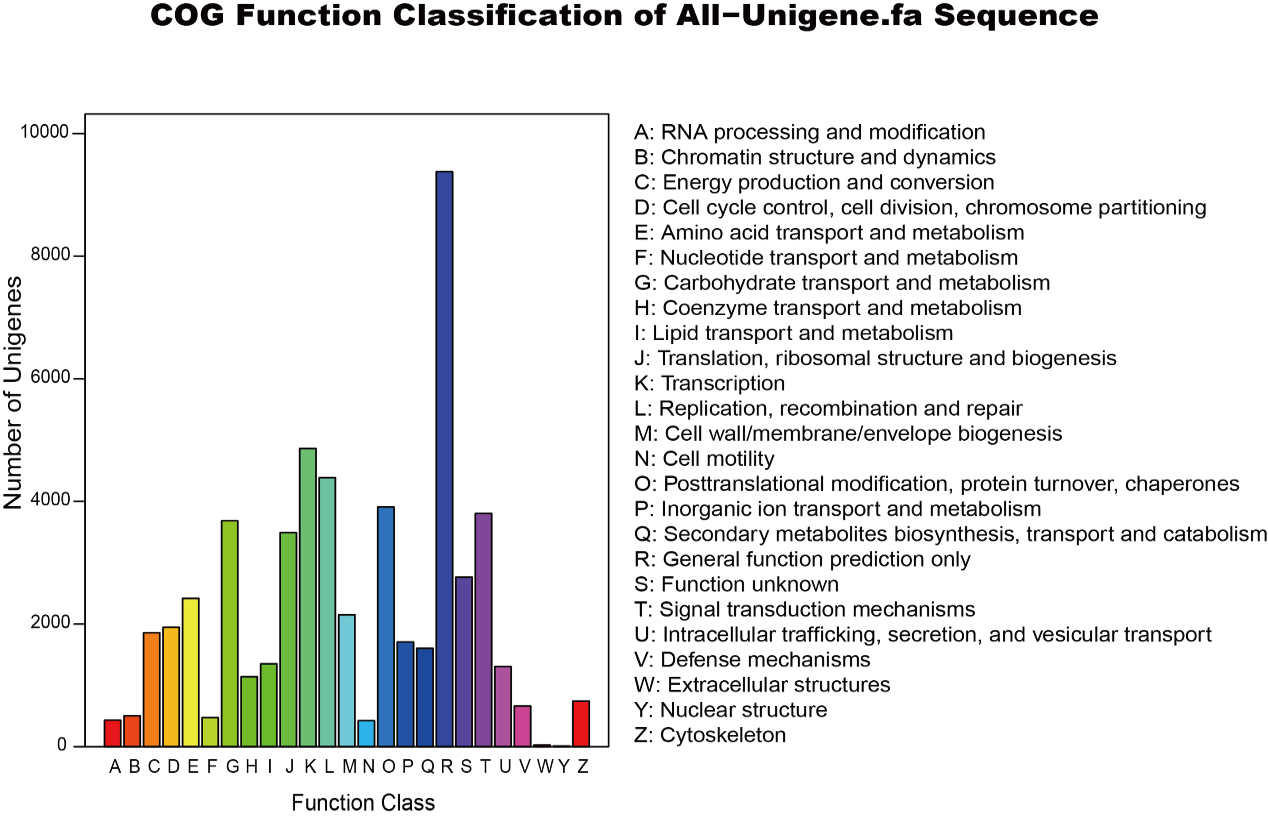
**

**Supplementary Figure S5.**

**
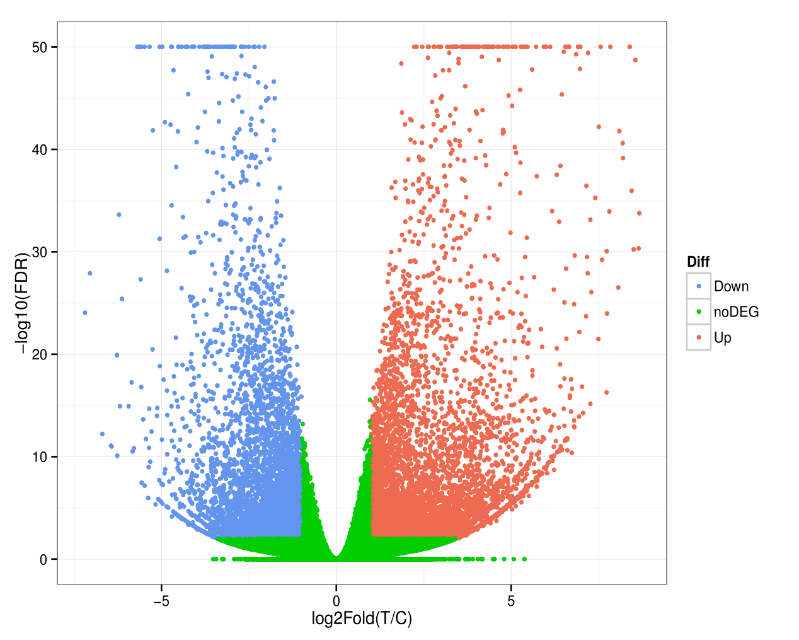
**

**Supplementary Figure S6.**

**
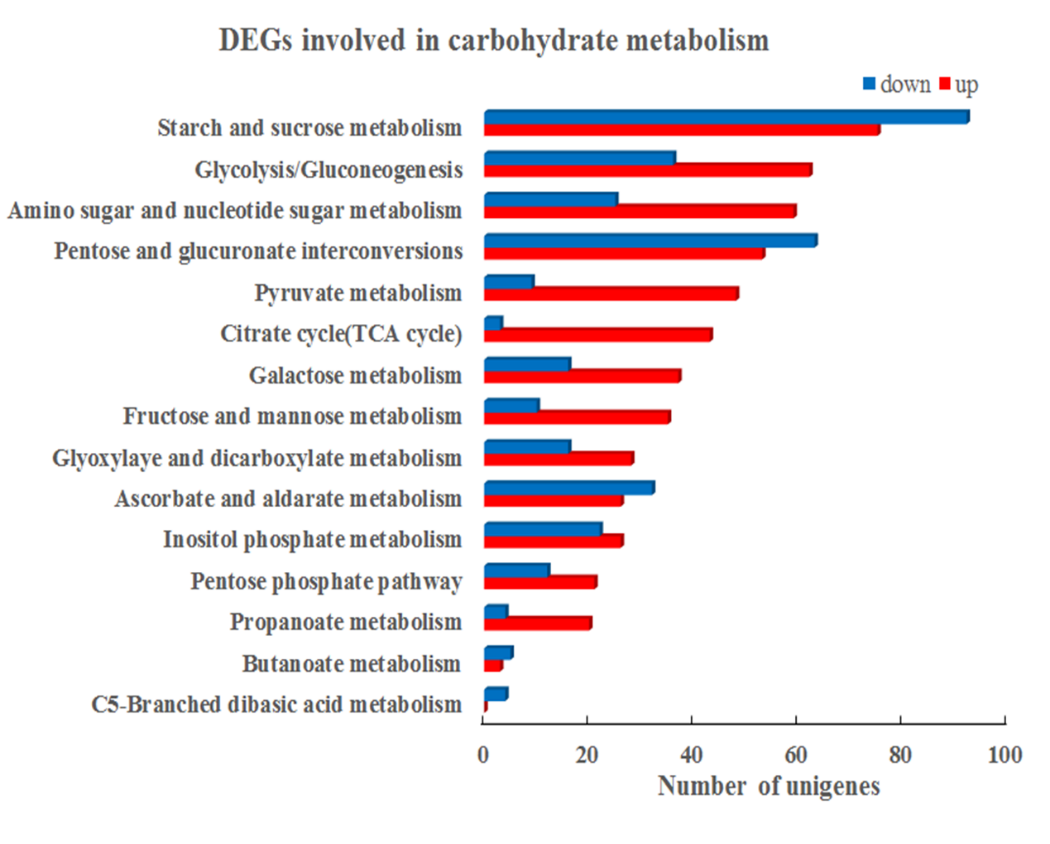
**

**Supplementary Figure S7.**

**
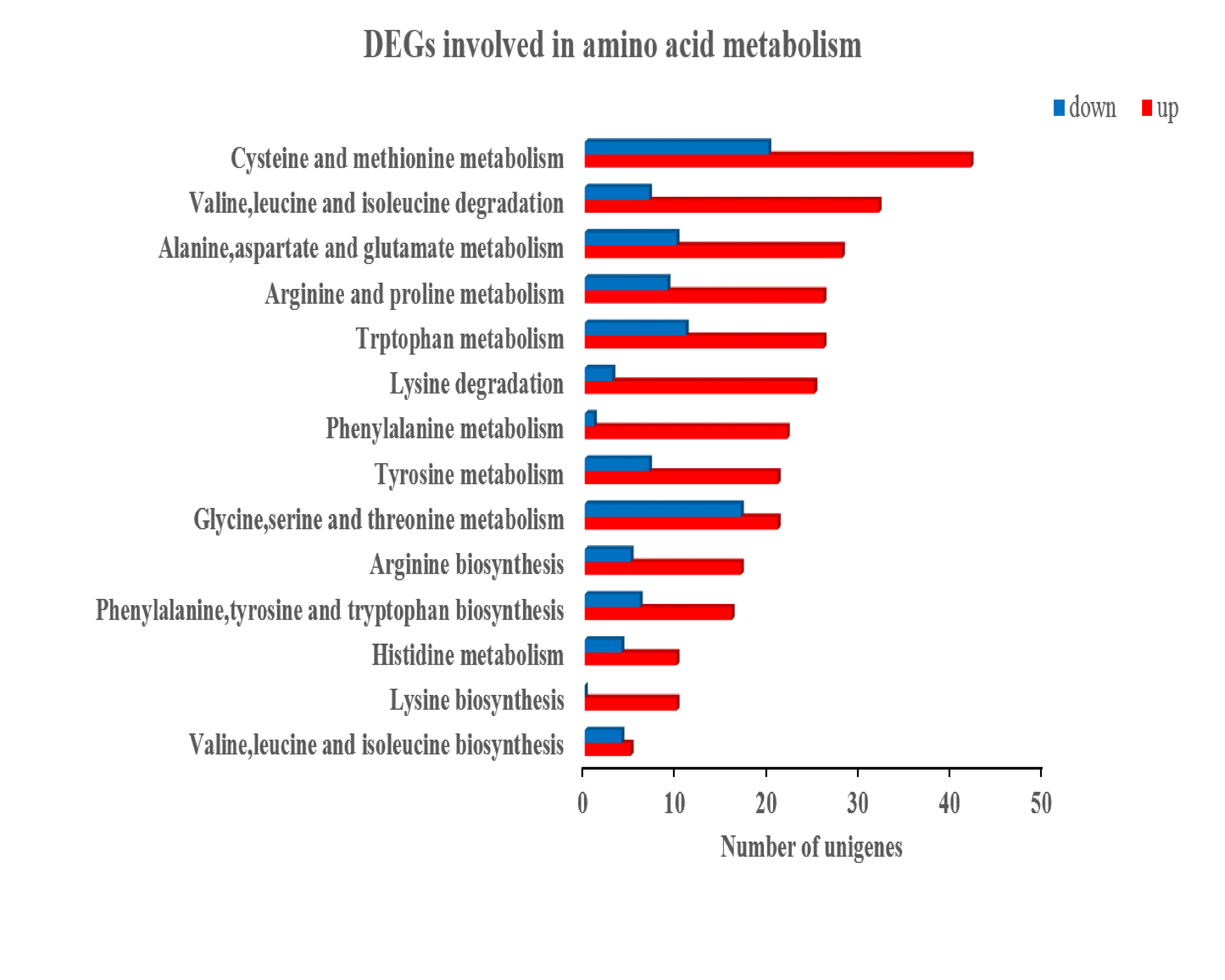
**
