# Supplementary material for: Comparative Transcriptome and Metabolic Profiling Analysis of Buckwheat (Fagopyrum Tataricum (L.) Gaertn.) under Salinity Stress
Source: Metabolites. 2019 Oct 14;9(10):225. doi: 10.3390/metabo9100225 (PMC6835380; doi:10.3390/metabo9100225)
Supplement: Supplementary file 1 [file metabolites-09-00225-s001.zip › Supplemental materials10-13/bw-nacl-supplementary file list-10-7.docx]

**Supplementary files**

**Supplementary Figure S1.** Length distribution of all unigenes from *F. tataricum* transcriptome.

**Supplementary Figure S2.** Annotation of all unigenes from *F. tataricum* transcriptome. Number of genes were annotated by five representatively databases (Nr, KEGG, COG, Swissprot, and Nt).

**Supplementary Figure S3.** GO analysis of all unigenes based on biological process, cellular component, and molecular function categories.

**Supplementary Figure S4.** COG function classification of all unigenes sequences from *F. tataricum* transcriptome.

**Supplementary Figure S5.** Distribution of differentially expressed genes (DEGs) between control and salt treated buckwheat. DEGs was selected by *p*-value < 0.001 and |log2FoldChange| > 2.

**Supplementary Figure S6.** DEGs involved in carbohydrate metabolism between control and salt treated buckwheat.

**Supplementary Figure S7.** DEGs involved in amino acid metabolism between control and salt treated buckwheat.

**Supplementary Table S1.** Statistics of annotations for assembled unigenes of *F. tataricum* in different public databases.

**Supplementary Table S2.** DEGs genes with KEGG pathway annotation.

**Supplementary Table S3.** The relative comparation of hydrophilic metabolites in control (Con) and salt-treated (Tr) buckwheat samples.

**Supplementary Table S4.** Table S4. The content of liphophilic metabolites in control (Con) and salt-treated (Tr) buckwheat samples.

**Supplementary Table S5.** Different expression of phenylpropanoid biosynthetic genes under salt stress.

**Supplementary Table S6.** The differentially expressed genes of carotenoids biosynthetic pathway in buckwheat under salt stress.

**Supplementary Table S7.** Identification of differentially expressed transcription factors (TFs) in buckwheat to response salt stress.
